# Characterization of Dysferlin Deficient SJL/J Mice to Assess Preclinical Drug Efficacy: Fasudil Exacerbates Muscle Disease Phenotype

**DOI:** 10.1371/journal.pone.0012981

**Published:** 2010-09-24

**Authors:** Sree Rayavarapu, Jack H. Van der meulen, Heather Gordish-Dressman, Eric P. Hoffman, Kanneboyina Nagaraju, Susan M. Knoblach

**Affiliations:** 1 Center for Genetic Medicine Research, Children's National Medical Center, Washington, D.C., United States of America; 2 Institute of Biomedical Sciences, The George Washington University, Washington, D.C., United States of America; 3 Department of Integrative Systems Biology, School of Medicine and Health Sciences, The George Washington University, Washington, D.C., United States of America; Brigham and Women's Hospital, Harvard Medical School, United States of America

## Abstract

The dysferlin deficient SJL/J mouse strain is commonly used to study dysferlin deficient myopathies. Therefore, we systematically evaluated behavior in relatively young (9–25 weeks) SJL/J mice and compared them to C57BL6 mice to determine which functional end points may be the most effective to use for preclinical studies in the SJL/J strain. SJL/J mice had reduced body weight, lower open field scores, higher creatine kinase levels, and less muscle force than did C57BL6 mice. Power calculations for expected effect sizes indicated that grip strength normalized to body weight and open field activity were the most sensitive indicators of functional status in SJL/J mice. Weight and open field scores of SJL/J mice deteriorated over the course of the study, indicating that progressive myopathy was ongoing even in relatively young (<6 months old) SJL/J mice. To further characterize SJL/J mice within the context of treatment, we assessed the effect of fasudil, a rho-kinase inhibitor, on disease phenotype. Fasudil was evaluated based on previous observations that Rho signaling may be overly activated as part of the inflammatory cascade in SJL/J mice. Fasudil treated SJL/J mice showed increased body weight, but decreased grip strength, horizontal activity, and soleus muscle force, compared to untreated SJL/J controls. Fasudil either improved or had no effect on these outcomes in C57BL6 mice. Fasudil also reduced the number of infiltrating macrophages/monocytes in SJL/J muscle tissue, but had no effect on muscle fiber degeneration/regeneration. These studies provide a basis for standardization of preclinical drug testing trials in the dysferlin deficient SJL/J mice, and identify measures of functional status that are potentially translatable to clinical trial outcomes. In addition, the data provide pharmacological evidence suggesting that activation of rho-kinase, at least in part, may represent a beneficial compensatory response in dysferlin deficient myopathies.

## Introduction

Limb Girdle Muscular Dystrophy type 2B (LGMD2B) is caused by the loss of function mutations in the dysferlin gene [1]. Dysferlin is primarily expressed in skeletal and cardiac muscle, but also in monocytes, macrophages, and other tissues where it is localized to cytoplasmic vesicles and the cell membrane [2], [3]. Dysferlin appears to be involved in membrane fusion and trafficking, as well as repair processes [4]. LGMD2B is a late onset (teens/young adults) muscle disease that is characterized by progressive symmetrical muscle weakness, and notably aggressive immune/inflammatory pathology. Muscle biopsies typically show marked inflammatory cell infiltration, consisting primarily of macrophages/macrophage activation markers (HLA-DR, HLA-ABC, CD86), CD8^+^ cytotoxic T cells, and CD4^+^ T cells, together with muscle fiber degeneration/regeneration [5], [6].

SJL/J mice have an in-frame deletion of 171 bp in the 3′ splice junction of exon 45 of dysferlin [7], [8]. They develop spontaneous myopathy that is associated with obvious muscle inflammation as they age. Together, these features make SJL/J mice a genetic homologue of human dysferlin deficient myopathies. Inflammatory changes in SJL/J mouse muscles typically begin around 4–6 weeks of age, and are characterized by infiltration of activated macrophages, followed by CD4+ T cells [8]–[10]. At 6 months, the infiltrate consists primarily of macrophages, along with some muscle fiber necrosis. By 16 months, muscle fibers completely degenerate and are replaced by fat and collagen [10], [11]. Although biochemical and histological features of the SJL/J strain have been relatively well documented, the functional and behavioral symptoms of the progressing myopathy have not been fully characterized, particularly during the early stages of disease, which are most likely to be useful for evaluation of potential treatment strategies.

We recently demonstrated that dysferlin-deficient monocytes from LGMD2B patients and SJL/J mice display enhanced phagocytic activity both in the unstimulated environment, as well as in response to inflammatory cytokines [4]. Expression of the Rho family GTPases RhoA, Rac1 and Cdc 42, which are involved in monocyte phagocytosis, are also increased in dysferlin-deficient immune cells relative to controls. In addition, markers of dendritic, T-cell, and macrophage activation (CD86, HLA-ABC, and HLA-DR) are expressed in LGMD2B muscle. These observations led us to propose that an overly aggressive inflammatory response in dysferlin deficient muscle may play a role in the progression of muscle disease [9]. Rho proteins are ubiquitous and have been widely studied for their potential contribution to many cellular functions, including motility and vesicular trafficking [12]–[14]. The consequences of Rho activation mainly depend on its downstream effectors, the Rho-kinases (ROCK) [15]–[16]. Rho-kinases exist as 2 isoforms: ROKα/ROCK II or ROKβ/ROCK I, which have many overlapping functions, although isoform specific differences are beginning to emerge. Activation of the ROCK pathway is a critical event in a number of inflammatory cell activities, including proliferation, phagocytosis, production of pro-inflammatory cytokines and in the activation of NF-kB [16]–[18]. Thus, inhibition of the ROCK pathway limits damage in models of inflammation, or in diseases that involve a significant inflammatory response component, such as asthma, sepsis, arthritis, and multiple sclerosis [19]–[22]. Indeed, the ROCK inhibitor fasudil has already been used clinically for several of these conditions. The major metabolite of fasudil, hydroxyfasudil, is an equipotent, relatively selective inhibitor of ROCKs -1 and -2, and shows 100 fold higher specificity for ROCK-2 than for protein kinase C or myosin light chain kinase [23], [24].

We recently determined which behavioral outcomes may be the most reliable for drug efficacy studies in Duchenne muscular dystrophy using mdx mice [25]. The present study undertakes a similar evaluation in the SJL/J mouse model of dysferlin deficiency. SJL/J mice were temporally evaluated and compared to C57BL6 control mice using several common behavioral measures, including grip strength (forelimb and hindlimb), open-field (digiscan) activity and rotarod performance. *In vitro* muscle force, serum creatine kinase, and histology were also examined. Further, we also performed a drug efficacy study with the Rho-kinase inhibitor fasudil, using these same parameters. Fasudil was selected for evaluation because of the established role of Rho- kinases in macrophage/monocyte activation and the potential involvement of these inflammatory cells in the progression of dysferlin deficient myopathies.

## Materials and Methods

### Experimental design

Six week-old male SJL/J (n = 30) and C57BL/6 mice (n = 30) weighing 20–25 g were obtained from the Jackson Laboratories (Bar Harbor, ME). All mice were housed in an individually vented cage system under controlled 12-h light/dark cycle with free access to feed and water. Animals were handled according to our Institutional Animal Care and Use Committee guidelines at Children's National Medical Center (Approved protocol # 214-07-01) and Washington DC Veterans Affairs Medical Center (Approved protocol # 1079). Each strain of mice was randomly separated into 2 groups, untreated (n = 15) and fasudil treated (n = 15) respectively. Untreated mice were given normal water, whereas fasudil treated mice were given fasudil *ad lib* via drinking water for 16 weeks at a dose of 75 mg/kg body weight starting at 9 weeks of age. This dosage is based on previously published oral dosages [20], [26], [27]. To verify the actual drug dosage, water consumption was assessed by measuring the amount of water consumed per cage, and dividing this by the number of animals present. Both treated and untreated SJL/J mice consumed more water (mean ± SE) (4.9±0.27 ml) per day, than did C57BL6 mice (3.3±0.08 ml), therefore, the actual concentration of fasudil in the water was adjusted based on the average water consumption for each strain. Treated mice in both strains initially drank less during the first week of treatment, but this resolved by the second week. Functional and behavioral data were obtained at 9, 17 and 25 weeks of age. The *in vitro* muscle function tests, serum creatine kinase levels, and histological evaluations were conducted at 25 weeks of age as described below.

### Functional tests

Grip strength was assessed using a grip strength meter (GSM) (Columbus instruments, Columbus, Ohio) as detailed previously [25]. All the mice were acclimatized on the GSM for 3 consecutive days, at least 1 week before actual data collection. Five successful forelimb and hindlimb strength measurements were recorded in the morning hours over a 5-day period by an investigator blinded to treatment. The maximum grip strength values of each day were used for subsequent analysis; the data were normalized to body weight and expressed as kilogram force/kg (KGF/kg).

Overall motor coordination was measured with a rotarod apparatus as described previously with slight modifications [25]. Mice were acclimatized on the rotarod (Ugo Basile, VA, Italy) for a period of four sessions for 2 days prior to the data collection. To perform the test, mice were placed on a rod rotating at a speed of 10 rpm. A maximum speed of 54 rpm was obtained within 4 minutes and the test was continued to a maximum of 3 minutes or until the mice fell from the rod. Data was collected by an investigator blinded to treatment twice a day, with a 2-h interval between the sessions, for 3 consecutive days. The time that a mouse stayed on the rod was recorded and the mean data were expressed as latency to fall (sec) for each mouse.

Open field behavioral activity was measured using an open field digiscan apparatus (Omnitech Electronics, Columbus, OH) as described previously [25]. All mice were acclimatized to the apparatus before actual data collection. The actual data were collected every 10 min over a 1-h period each day for 4 consecutive days and the results were expressed as mean ± SE for all behavioral parameters [25].

### Histological evaluation

All mice were sacrificed at 25 weeks of age, and the tibialis anterior (TA) and quadriceps femoris muscles were collected for hematoxylin and eosin staining (H&E) and immunohistochemistry, respectively. The muscles were kept in formalin for H&E staining or embedded in Tissue-Tek (West Chester, PA) and flash frozen in liquid nitrogen chilled isopentane for immunohistochemistry. Serum was also acquired from these mice to estimate the creatine kinase levels. For hemotoxylin and eosin staining, the TA of fasudil treated (*n* = 5) and untreated (*n* = 5) mice were collected and stained as previously described [25]. For quantification, 5 non-overlapping representative fields of the stained tissue sections were imaged under a light microscope (40X) and a digital image for each field was obtained using computer software (Olympus America Inc., Center Valley, PA). The digital images were loaded into Image J (NIH) with an additional plug-in to count cells. Briefly, total fibers present; total fibers with central nuclei; regenerating fibers (basophilic fibers); degenerating fibers; and inflammation (defined as an interstitial group of >10 smaller inflammatory cell dark blue nuclei in a high power field) were assessed. Quantitative procedures were performed as detailed previously [25].

To determine the change in the number of infiltrating macrophages the frozen sections of quadriceps femoris muscle from fasudil treated (*n* = 4) and untreated (*n* = 4) SJL/J mice were stained with rat anti-mouse F4/80 antibody (Serotec, Oxford, UK). F4/80 antibody served as a marker for infiltrating monocytes, and macrophages. Anti-rat horseradish peroxidase (DAKO, Carpinteria, CA) was used as a secondary antibody. Isotype-matched mouse immunoglobulins were used as negative controls. Three non-overlapping fields of the entire tissue section were imaged (20X) and quantified for the presence of inflammatory foci. The foci were defined as aggregates of F4/80^+^ macrophages and monocytes.

### *In vitro* force measurement

The EDL and soleus muscles of the right hindlimbs were removed from anesthetized mice, and were placed vertically in a bath containing buffered mammalian Ringer solution (25°C) and bubbled with 95% O_2_–5% CO_2_. The distal tendon of the muscle was tied securely to the lever arm of a servomotor/force transducer (model 305B, Aurora Scientific) and the proximal tendon was fixed to a stationary post in the bath. The muscles were stimulated between two stainless steel plate electrodes. At optimal muscle length, the force developed was measured during trains of stimulation (1000 ms for soleus, and 300 ms for EDL muscles) with increasing frequencies till the highest plateau was achieved. The force generated to obtain the highest plateau was established as the maximal force generated by the muscle and is expressed in milli Newton (mN). Further, the specific force was obtained by dividing the maximal force with the physiologic cross sectional area of muscle and is expressed as kN/m^2^. The physiologic cross sectional area was calculated by dividing the muscle mass with the fiber length and density (1.056 kg/m^3^) of muscle tissue. The fiber length is established as 0.45 and 0.71 of muscle length for the soleus and EDL muscles respectively [28].

### Creatine kinase determination

Blood (250 µl) was collected into eppendorf tubes by cardiac puncture immediately after euthanasia and was allowed to clot at room temperature prior to centrifugation and serum collection. Creatine kinase (CK) determination was performed according to the manufacturer's instructions using a standard spectrophotometric method [29], [30]. Absorption at 340 nm was measured every minute for 2 minutes at 37°C to calculate enzyme activity. Duplicate measurements were done on each serum sample, and the data were expressed as units per liter.

### Statistical analysis

As the behavioral data were not normally distributed we performed non-parametric tests for the statistical analysis. The drug effects (drug vs untreated controls) of each strain type were compared at each time point using the Wilcoxon rank sum test. To assess the effect of drug treatment and time on individual behavioral outcomes over the 9–25 week study period, the raw data for each test were ranked and then 2-way repeated measures ANOVA with factor A as drug and factor B as time was used for statistical assessment. The histological and *in vitro* force measurements were compared between treated and untreated wild-type or SJL/J mice at each time point using student's *t*-test for independent samples. A value of p<0.05 was considered significant. Power values were determined using estimates from each mouse strain and assuming an *N* of 10 per group. The effect size necessary to detect a significant association with an *N* of 10 was determined by using C57BL6 as the reference group and calculating the effect size that would produce a significant difference.

## Results

### Phenotypic differences between dysferlin deficient SJL/J and C57BL6 control mice

SJL/J mice generally weighed less than C57BL6 mice at all ages and they gained less weight overall during the course of the study (C57BL6 vs SJL/J: mean ± SEM 11.17±0.73 vs 5.59±0.59; p<0.0001, Wilcoxon Rank Sum test) (Table 1). Since body weight may influence grip strength, the grip strength data were normalized to body weight. Thus the normalized forelimb (NFL) grip strength was greater in SJL/J mice at all ages than in C57BL6 mice (Table 1). However, both strains showed proportional decreases in NFL grip strength over the course of the study. Normalized hindlimb (NHL) grip strength was also higher in SJL mice than in C57BL6 mice at older ages (17 and 25 weeks). There were no significant differences in rotarod performance between mouse strains (Table 1).

**Table 1:** Comparison of weight, grip strength, and rotarod performance of C57BL6 and SJL/J mice at 9, 17 and 25 weeks of age.

Measurement	Strain	9 weeks		17 weeks		25 weeks		Overall p-value*	Significantly different means
		N^1^	Mean ± SEM	N^1^	Mean ± SEM	N^1^	Mean ± SEM		
Weight (gm)	C57BL/6	15	21.87±0.31 a,b	14	29.47±0.69 a,c	14	32.96±0.91 b,c	<0.0001	a p<0.001^b^ p<0.001^c^ p = 0.002
	SJL/J	15	20.60±0.21 a,b	13	22.87±0.49 a,c	13	26.13±0.55 b,c	<0.0001	a p = 0.002^b^ p<0.001^c^ p<0.001
NFL^2^	C57BL/6	15	4.81±0.08 a,b	14	3.31±0.10 a	14	3.24±0.12 b	<0.0001	a p<0.001^b^ p<0.001
	SJL/J	15	6.19±0.24 a	13	6.73±0.31 b	13	5.06±0.26 a,b	0.0004	a p = 0.014^b^ p<0.001
NHL^2^	C57BL/6	15	11.39±0.17 a,b	14	8.98±0.25 a	14	8.88±0.30 b	<0.0001	a p<0.001^b^ p<0.001
	SJL/J	15	10.83±0.18 a	13	14.25±0.47 a,b	13	11.00±0.41 b	<0.0001	a p<0.001^b^ p<0.001
Rotarod (s)^3^	C57BL/6	15	98.8±10.2	14	79.0±8.5	14	70.4±7.8	NS^4^	
	SJL/J	15	88.9±7.4	13	73.5±6.7	13	87.3±7.3	NS^4^	

All data are expressed as mean ± SE,

1 Number of animals per group,

2 NFL (Forelimb) and NHL (Hindlimb) grip strength data are normalized to body weight and are expressed in kilogram force per kilogram (KGF/kg),

3 Rotarod, the length of time in seconds that mice stayed on the rotarod.

* *p*-values are from Wilcoxon rank sum test at each time point,

4 NS, not statistically significant.

a, b, c indicate the p value for the significant differences between means with the same letter (eg a vs a etc.).

A number of behavioral differences between SJL/J and C57BL6 mice were detected in the open field measurements (Table 2). In these tests, SJL/J mice performed significantly worse than C57BL6 mice by nearly all measures. They displayed less horizontal or vertical activity, traveled a shorter total distance, and spent more time resting in the open field than did C57BL6 controls. Moreover, for SJL/J mice, scores on all these measures were significantly lower at 25 wks than at 9 wks, whereas scores for C57BL6 mice were unchanged over time. Thus, all these tests detected deterioration in performance in the SJL/J mice. All the open field measurements had relatively high power, although effect sizes varied widely across specific outcomes. Rest time appears to have been the most sensitive indicator as reflected in an effect size of 2%–3.3% (Table 2).

**Table 2:** Comparison of open field behavioral activity of C57BL6 and SJL/J mice at 9, 17 and 25 weeks of age.

Measurement	Age (wk)	N^1^	C57BL/6Mean ± SE	N^1^	SJL/JMean ± SE	*p*-value*	Power with N = 10 per group	Effect size necessary with N = 10 per group∧
Horizontal activity	9	15	885.1±55.7	15	523.5±19.4	<0.0001	99.9%	32.3%
	17	14	882.6±74.3	13	389.8±31.4	<0.0001	99.9%	41.8%
	25	14	1008.0±86.0	13	405.5±19.1	<0.0001	99.9%	42.3%
Total distance (cm)	9	15	194.8±19.5	15	85.9±8.0	<0.0001	98.8%	51.1%
	17	14	152.9±25.5	13	32.9±5.5	0.0002	97.4%	82.7%
	25	14	185.0±33.0	13	39.3±11.3	0.0004	94.3%	88.5%
Rest time (s)	9	15	576.4±2.2	15	590.6±0.9	<0.0001	99.8%	2.0%
	17	14	581.4±3.1	13	595.8±0.7	0.0002	97.5%	2.6%
	25	14	577.8±3.8	13	595.6±1.2	0.0002	96.7%	3.3%
Vertical activity	9	15	20.5±2.2	15	7.6±1.0	<0.0001	99.3%	54.3%
	17	14	15.7±1.8	13	3.3±0.9	<0.0001	99.9%	55.9%
	25	14	19.4±2.9	13	3.2±0.8	<0.0001	99.6%	73.9%

All data are expressed as mean ± SE;

1 Number of animals per group;

* *p*-values are from Wilcoxon rank sum at each time point;

∧ Effect size between C57BL/6 and SJL/J mice that is necessary to conclude a significant difference.

Weights of selected muscles, and the heart and spleen of C57BL6 controls and SJL/J mice were measured at 25 weeks of age (Table 3). Notably, the weight of the Soleus in SJL/J mice was half that observed in C57BL6 mice, whereas the weight of the SJL/J spleen was nearly double that of C57BL6 mice. No significant differences were observed in the weights of extensor digitorum longus (EDL) or tibialis anterior muscle.

**Table 3:** Weights of muscles, heart and spleen of C57BL6 and SJL/J mice at 25 weeks of age.

Name	C57BL6		SJL/J		*p*-value* (Weight)	*p*-value# Normalized
	Weight^1^	Normalized^2^	Weight^1^	Normalized^2^		
Soleus	10.47±0.74	0.33±0.02	5.06±0.14	0.19±0.01	<0.0001	<0.0001
EDL^3^	12.44±1.04	0.39±0.03	10.59±0.19	0.39±0.01	0.1174	0.9138
TA^4^	57.11±4.60	1.79±0.13	53.37±0.86	1.99±0.06	0.4649	0.2040
Gastrocnemius	141.00±4.66	4.45±0.14	137.60±2.37	5.10±0.09	0.5452	0.0010
Heart	130.20±3.65	4.10±0.08	139.30±3.65	5.17±0.15	0.0929	<0.0001
Spleen	76.46±1.70	2.42±0.07	170.70±6.36	6.28±0.56	<0.0001	<0.0001

All data are expressed as mean ± SE;

1 Raw weight of the muscle expressed in milligrams (mg);

2 Raw weight of muscle normalized to respective bodyweight;

3 Extensor digitorum longus;

4 Tibialis anterior;

* *p*-values for student's *t*-test comparison of raw muscle weights between C57BL6 and SJL/J mice;

# *p*-values for student's *t*-test comparison of normalized weights between C56BL6 and SJL/J mice.

**Figure 1:**
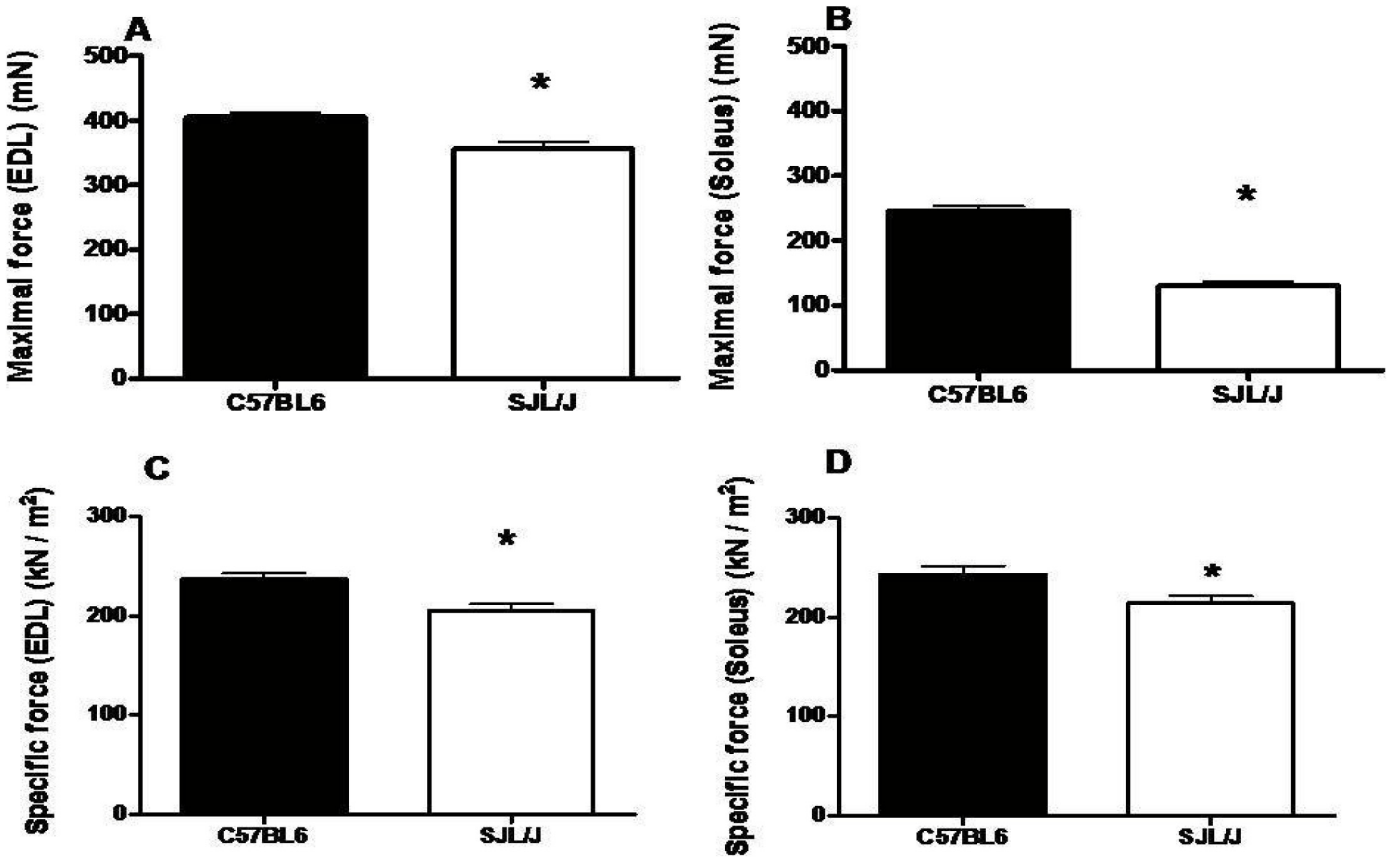
Maximal and specific force of extensor digitorum longus (EDL) and soleus muscles of SJL/J and C57BL6 mice: At 25 weeks of age SJL/J (*n* = 5/group) and C57BL6 (*n* = 8/group) mice were sacrificed and *in vitro* muscle function tests were performed for EDL (A, C) and soleus (B, D) muscles. Shown are the values obtained for maximal force (mN) (A & B), specific force (kN/m^2^) (C & D). Values were compared using Student's *t*-test for independent samples, where * = *p*<0.05.

*In vitro* force contractions were measured in isolated EDL and Soleus muscles of SJL/J and C57BL6 mice at 25 weeks of age. The maximal and specific force of both muscles was significantly less in SJL/J mice compared with C57BL6 (Figure 1). The mean CK value (U/L) of SJL/J mice (826.0±135.9) was higher (*p*<0.05) than in C57BL6 mice (378.8±99.21).

### Effect of fasudil treatment on the behavioral phenotype(s) of dysferlin deficient (SJL/J) and control (C57BL6) mice

Treatment with fasudil decreased both the NFL and NHL grip strength of SJL/J mice compared to untreated controls (Table 4). Although only the 17 wk NFL individual time comparison between treated and untreated is statistically different by the Wilcoxon rank sum test, the 2-way ANOVA comparison of all times in treated vs. untreated SJL/J mice indicated a significant effect of both drug (*p* = 0.0278) and time (*p*<0.0001) on NFL performance between the 2 groups from 9 to 25 wks of age. Similarly, NHL scores were significantly less at both 17 and 25 wks in fasudil treated vs untreated SJL/J controls, and the 2-way ANOVA comparison for NHL scores of all times in treated vs untreated SJL/J mice indicated significant effects of both drug (*p* = 0.0002) and time (*p*<0.0001) from 9 to 25 wks of age. Fasudil did not have any effect on rotarod performance in SJL/J mice (Table 4).

**Table 4:** Comparison of weight, grip strength, and rotarod performance of fasudil treated and untreated SJL/J mice at 9, 17, and 25 weeks of age.

Measurement	Age (wk)	N^1^	TreatedMean ± SEM	N^1^	UntreatedMean ± SEM	*p*-value*	Power with N = 10 per group
Weight (gm)	9	15	20.53±0.34	15	20.60±0.21	NS^2^	5.2%
	17	12	24.44±0.41	13	22.87±0.49	0.0179	59.0%
	25	11	26.81±0.67	13	26.13±0.55	NS^2^	11.1%
NFL^3^	9	15	6.08±0.19	15	6.19±0.24	NS^2^	6.2%
	17	12	5.74±0.23	13	6.73±0.31	0.0339	63.2%
	25	11	4.32±0.15	13	5.06±0.26	NS^2^	59.5%
NHL^3^	9	15	10.52±0.25	15	10.83±0.18	NS^2^	13.2%
	17	12	12.17±0.41	13	14.25±0.47	0.0065	84.2%
	25	11	9.69±0.29	13	11.00±0.41	0.0257	66.2%
Rotarod (s)^4^	9	15	90.6±6.2	15	88.9±7.4	NS^2^	5.3%
	17	12	75.9±5.1	13	73.5±6.7	NS^2^	5.8%
	25	11	68.2±8.7	13	87.3±7.3	NS^2^	34.4%

All data are expressed as mean ± SEM;

1 Number of animals per group;

2 NS, not statistically significant;

3 NFL (Forelimb) and NHL (Hindlimb) grip strength data are normalized to body weight and are expressed in kilogram force per kilogram (KGF/kg);

4 Rotarod, the length of time in seconds that mice stayed on the rotarod;

* *p*-values are from Wilcoxon rank sum tests at each time point.

While most of the open field tests showed no effect of fasudil treatment on outcome, all the tests indicated a significant decline in performance over time in both fasudil treated and untreated SJL/J controls (2-way ANOVA for all tests, effect of time, *p*<0.0001) (Table 5). The only open field test to show a significant negative effect of fasudil was the horizontal activity score, which indicated that fasudil treatment resulted in significantly less horizontal activity compared to untreated SJL/J controls at 25 wks of age.

**Table 5:** Comparison of open field behavioral activity of fasudil treated and untreated SJL/J mice at 9, 17, and 25 weeks of age.

Measurement	Age (wk)	N^1^	TreatedMean ± SEM	N^1^	UntreatedMean ± SEM	Power with N = 10 per group
Horizontal activity	9	15	549.8±36.4	15	523.5±19.4^a^	8.0%
	17	12	358.8±24.9	13	389.8±31.4^a^	10.6%
	25	11	306.5±32.1	13	405.5±19.1^b^	69.4%
Total distance (cm)	9	15	101.3±11.7	15	85.9±8.0^a^	14.3%
	17	12	29.8±4.2	13	32.9±5.5^a^	6.8%
	25	11	19.2±3.4	13	39.3±11.3^a^	32.1%
Rest time (s)	9	15	589.0±1.3	15	590.6±0.9^a^	15.0%
	17	12	596.3±0.5	13	595.8±0.7^a^	6.5%
	25	11	597.5±0.5	13	595.6±1.2^a^	29.4%
Vertical activity	9	15	10.2±1.8	15	7.6±1.0^a^	16.8%
	17	12	3.3±0.7	13	3.3±0.9^a^	5.0%
	25	11	1.5±0.5	13	3.2±0.8^a^	39.1%

All data are expressed as mean ± SEM;

1 Number of animals per group;

a Indicates no significant difference between treated and untreated groups;

b Indicates significant difference between treated and untreated groups (Wilcoxon rank sum test, p = 0.0138).

There was little evidence to suggest that fasudil treatment worsened outcomes in C57BL6 mice as it did in the SJL/J strain (Supplementary Tables S1 and S2). While fasudil treatment did result in significantly worse NHL grip strength compared to untreated controls at 25 wks of age, it also increased body weight at this time point. Moreover, it improved horizontal activity scores not just at 17 wks, but more importantly, overall (2-way ANOVA for 9–25 wks of age, drug effect (p = 0.01). The significant difference between treated and untreated at 9 wks does not reflect a drug effect, because the 9 wk score is the baseline score obtained before initiation of treatment. Fasudil appeared to increase distance traveled and vertical activity in the open field, as well as reduce rest time, however such changes were only observed at 17 wks of age, rather than over the entire study (9–25 wks), or at both 17 and 25 wks. The reasons for this change in behavioral activity at 17 wks are presently unclear.

### Effect of fasudil treatment on muscle force, histology and inflammation in dysferlin deficient (SJL/J) and control (C57BL6) mice

**Figure 2:**
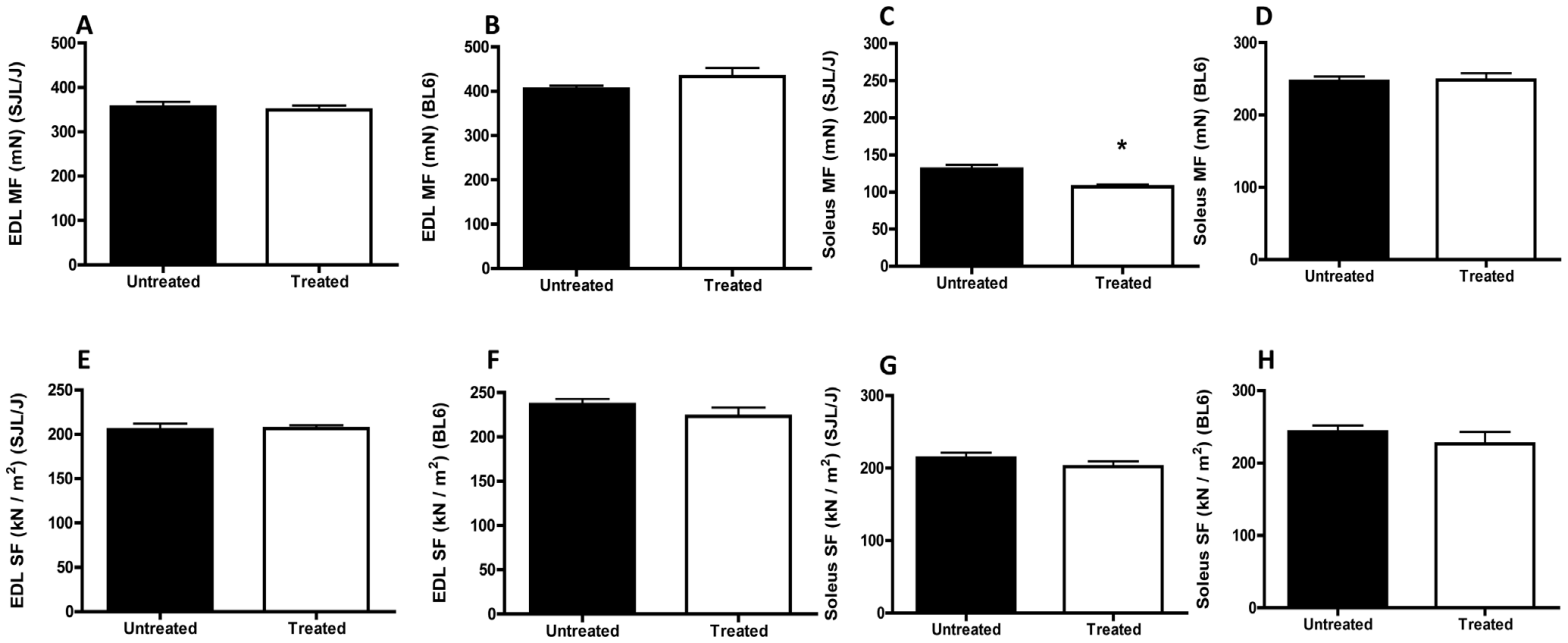
Effect of fasudil on extensor digitalis longorum (EDL) and soleus muscle force: At 25 weeks of age, *in vitro* muscle function tests were performed on EDL (A, B, E, F) and soleus (C, D, G, H) muscles to determine the affect of fasudil treatment on muscle force in C57BL6 (B, F, D, H) and SJL/J (A, E, C, G) mice. Shown are the graphs for maximal force (MF) (mN) (A–D) and specific force (SF) (kN/m^2^) (E–H) for soleus and EDL of each strain. Treatment groups of each strain were compared using student's *t*-test for independent samples. *p = <0.05 by Student's t-test comparisons.

Fasudil treatment did not alter the maximal (Figure 2, A and B) or specific (Figure 2, E and F) force of EDL muscle in either mouse strain. However, fasudil treatment significantly reduced the maximal force of soleus muscle in SJL/J mice (Figure 2, C), but had no effect on the maximal force of soleus muscle of C57BL6 mice (Figure 2, D). No significant differences were observed in the specific force of soleus muscle between treated or untreated groups of either strain (Figure 2G and H). The mean ± SEM CK values of fasudil treated and untreated SJL/J mice were 1056±238.8 and 826.0±135.9, respectively. However, these differences were not statistically significant.

There was no difference in the mass of the spleen between untreated or fasudil treated mice in either the SJL/J or C57BL6 strains. Values (mean ± SE) for the normalized spleen mass for SJL/J untreated and fasudil treated groups were 6.28±0.56 and 6.59±0.73 respectively. The normalized spleen mass for C57BL6 untreated and fasudil treated groups were 2.42±0.07 and 2.31±0.11 respectively.

**Figure 3:**
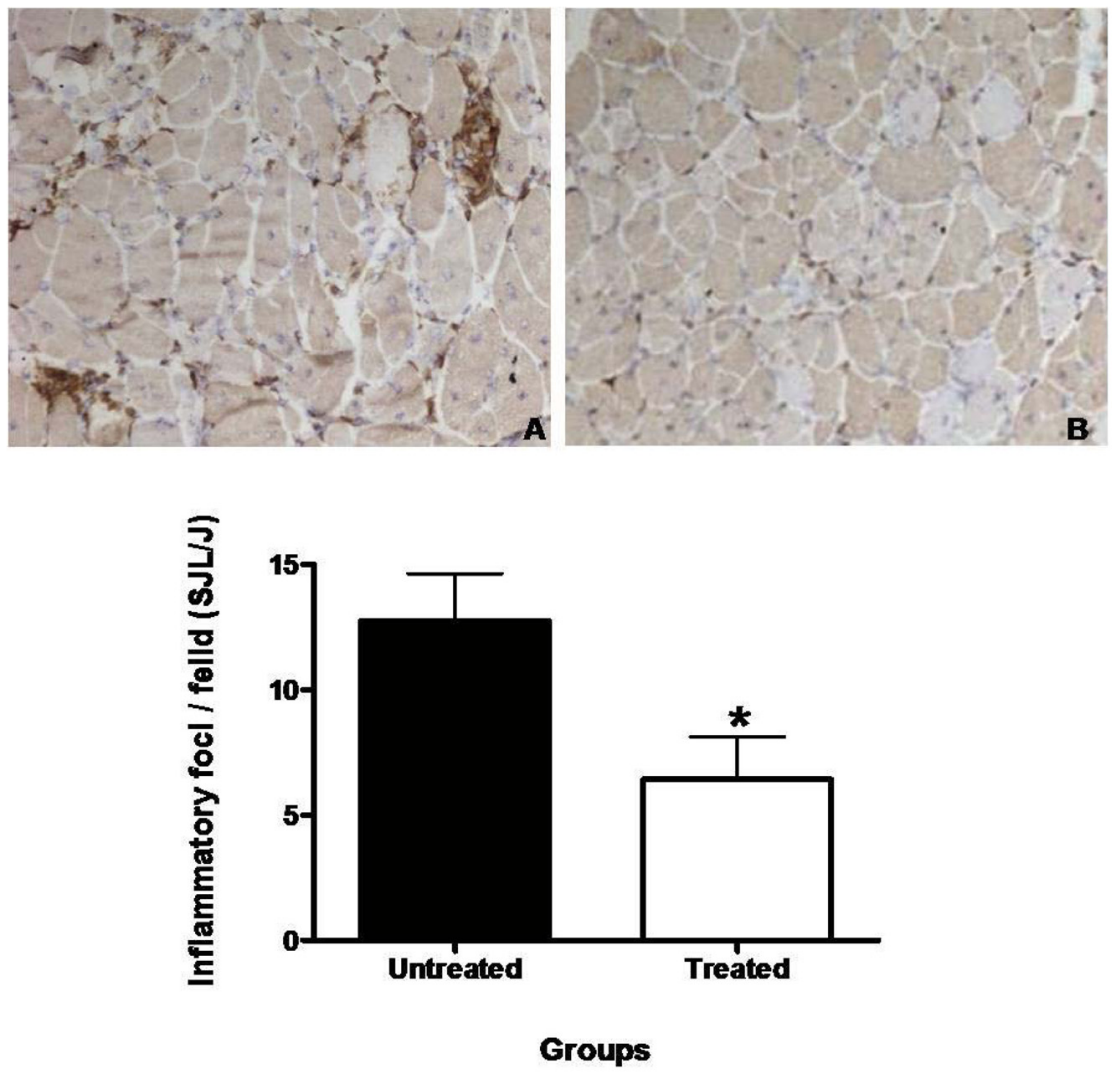
Effect of fasudil on inflammatory foci: Representative sections of untreated (A) and fasudil treated (B) (*n* = 4/group) quadriceps femoris muscle of SJL/J mice sacrificed at 25 weeks of age. Frozen sections of quadriceps femoris were stained with rat anti-mouse F4/80 (Serotec, Oxford, UK) primary antibody. Anti-rat horse-radish peroxidase (DAKO, Carpinteria, CA) was used as a secondary antibody. Three non-overlapping fields of the entire tissue section were imaged (20X) and quantified. Data were graphed (mean ± SEM) and the groups were compared using student's *t*-test for independent samples, where *p<0.05 by Student's *t*-test comparison.

Fasudil treatment did not significantly change a number of histological features observed in the TA muscle of SJL/J mice (supplementary Table S3). For example, fasudil did not alter the total number of muscle fibers, the number of degenerating or regenerating fibers, or the number of central nuclei. However, the quadriceps femoris of fasudil treated SJL/J mice displayed a significant reduction in the number of inflammatory foci compared to untreated SJL/J mice (Figure 3). There was no notable difference in the location of inflammatory infiltrates between treated vs control animals. Histological evaluation was not performed for C57BL6 mice.

## Discussion

This study assessed phenotyping and preclinical evaluation methods for dysferlin deficient SJL/J mice, focusing on behavioral (grip strength, open field, and rotarod), functional, (*in vitro* force measurements on isolated muscle) and histological measurements. Significant differences in body weight, grip strength, behavioral activity and *in vitro* force contraction measurements between C57BL6 and SJL/J mice were observed. Moreover, a progressive deterioration of functional performance in SJL/J mice was detectable from 9 to 25 weeks of age, which is relatively early in the development of spontaneous myopathy. Treatment with the rho-kinase inhibitor fasudil did not ameliorate the dysferlin deficient phenotype. Rather, fasudil treatment resulted in reduced grip strength (both fore limb and hind limb) and reduced horizontal activity. In contrast, fasudil treatment of C57BL6 mice either had no effect on behavioral outcomes, or improved them. The maximal force of soleus muscle was reduced in SJL/J mice treated with fasudil, but the drug had no effect on muscle force in C57BL6 mice. Lastly, fasudil treated SJL/J mice had fewer F4/80 labeled macrophages/monocytes in skeletal muscle, although other muscle histology parameters were unchanged.

Evaluation of outcome measures to use in preclinical studies is an important step toward developing standardized testing procedures for drug trials of new therapies for the treatment of muscular dystrophies. As preclinical outcomes for mdx mouse trials were recently assessed [25], the present paper focuses on a similar standardized evaluation of commonly used behavioral tests in dysferlin deficient SJL/J mice, which have been proposed as an animal model for dysferlin deficient myopathies [8]. The genomic and histological features of SJL/J mice are fairly well described [7], [9], [10], [31]. In addition, muscle and trunk weakness have been observed in SJL/J mice as early as 3 wks of age, when they are unable to extend their limbs upon suspension by the tail [8]. However, this is the first systematic review of their functional status, particularly with regard to power analysis for preclinical pharmacological studies. Functional deficits were detected in young SJL/J mice that otherwise have no overt symptoms of disease. Moreover, drug effects were detected as early as 17 wks of age, and such effects may possibly be detected even earlier, as significant differences between SJL/J and C57BL6 mice were apparent as early as 9 wks of age.

Normalized grip strength was a very sensitive indicator of function and drug effect. Normalized grip strength measurements have been routinely used to assess disease phenotype as well as therapeutic efficacy of various agents in the dystrophin deficient mdx mice [25], [30], [32]. The effect size calculations in this study indicate that, as with mdx mice, normalized grip strength is a reliable outcome measure to study the changes in the disease phenotype of SJL/J mice. Despite their muscle disease, normalized forelimb grip strength of the SJL/J mice was significantly greater than C57BL6 mice at all ages tested (Table 1). This finding is consistent with a report that showed the strength of young SJL/J mice was significantly more than Balb/c mice [10]. The reasons for increased grip strength in dysferlin deficiency are not clear. Nevertheless, SJL/J mice showed a significant reduction in normalized grip strength (both fore and hindlimb) between 17 and 25 weeks of age, as also previously observed [10]. This change in relative strength may be due to the decrease in grip strength which reflects the progression of the underlying muscle disease, but also related to the increased weight of the SJL/J mice over time.

Open field measures were among the most sensitive outcome measures in the SJL/J mouse. This is an important observation since open field activity tests are relevant to the timed walk test in humans with neuromuscular disease, which is common clinical trial endpoint. We observed a significant decrease in the vertical activity, horizontal activity, and total distance traveled in SJL/J compared with C57BL6 mice (Table 2). This is correlated to the significant increase in the rest time in SJL/J compared with C57BL6 mice. Notably however, there is significant variability in the sensitivity of different open field parameters, which is reflected in different effect sizes for horizontal activity, total distance and vertical activity (34%–89%).

The rotarod was a relatively insensitive test. The latter is surprising since different rota-rod protocols have been used successfully with the SJL/J strain in studies of experimental autoimmune encephalomyelitis, and routinely in studies of motor neuron degeneration in the B6SJL-Tg (SOD1G93A) 1Gur/J mouse, but both of these have manipulations that induce much stronger disease phenotypes (E.g., complete paralysis), than observed in SJL/J mice [33], [34]. Thus, it is possible that the rota-rod test is not as sensitive to subtle behavioral defects, as are some of the other measures.

In the present study, the assessments were performed every 8 wks over a 17 wk period-more frequent measurements may develop a more accurate assessment of subtle functional changes over time, and detect session-related outliers better. Nevertheless, more frequent testing will also raise issues with behavioral adaptation of mice to the testing environment. Thus more work is needed to determine the optimal testing schedule for assessments of SJL/J behavior. The present study also evaluated muscle force, serum creatine kinase and weights of selected muscles as measures of SJL/J myopathy. Collectively, the reduced muscle force and increase in creatine kinase observed in SJL/J mice relative to C57BL6 controls at 25 wks of age suggest ongoing muscle pathology. These findings are consistent with a previous report that observed a significant reduction in the specific force of the tibialis anterior muscle of C57BL/10.SJL-Dysf mice compared to normal mice [35]. Nevertheless, such degeneration may evolve selectively, since the soleus and heart muscles in the present study seemed to be preferentially affected at 25 wks of age. Indeed, cardiomyopathy has been previously described in other models of dysferlin deficiency [36]. Elevations of serum CK have also been observed in dysferlin deficient mice, and are associated with increasing age [9]. While elevations in serum CK are consistent with dysferlin deficiency, serum CK levels in our study and others have large variation, and thus tend to be less useful for small studies.

The increased spleen weight of SJL/J mice compared to C57BL6 controls may be associated with the immune/inflammatory dysregulation of these mice, as they have elevated levels of circulating T cells and are prone to lymphomas with aging [37]. It is not clear how or even if, these SJL/J characteristics are related to dysferlin deficiency. Notably, the SJL/J strain has other gene mutations; in addition to the mutation in dysferlin gene (www. Jax.org). SJL/J strain also has a unique susceptibility to autoimmune dysfunction, which makes it useful for models of multiple sclerosis, encephalitis, myositis, and colitis [38]–[41]. It also serves as a background strain in the B6SJL-Tg (SOD1G93A)1Gur/J model of amyotrophic lateral sclerosis, which has a faster progressing form of motor neuron disease than that found in other mouse strains with the same SOD1 mutation. Moreover the faster rate of disease progression in this strain is not linked to the dysferlin deficiency [42]. It is important to recognize these caveats when attempting to draw associations between the pathobiology of this strain and that of dysferlin deficient myopathies. Because the SJL/J mouse is a unique inbred strain, there is no matched control strain for it, although swiss-derived strains (SWR/J or FVB/J) are probably the closest genetically (www.jax.org). However, C57BL/6 was used as a control strain in the present work because it is the most widely used general purpose inbred strain, and is commonly used as a background strain for generation of congenics carrying spontaneous or induced mutations, as well as for transgenics.

Overall, based on the data from the tests evaluated here, we suggest that open field and normalized grip strength are probably the best outcome measures to use for preclinical drug testing in the SJL/J mice The open field test may be particularly useful, as it is not likely to be as sensitive to behavioral adaptation (especially if animals are conditioned to their environment beforehand) as some of the other tests, and is more amenable to standardization across laboratories. In addition, the behavioral parameter such as the total distance (cm) is similar to clinical correlate such as 6 minute walk test that is used to evaluate drug efficacy human dystrophy clinical trials.

Both the histopathological data and the behavioral data for grip strength correlate with other reported results for SJL/J strain. For example Weller et al., reported 14% reduction in the mean strength of SJL/J mice between 4 and 6 months of age. The grip strength of SJL/J mice in our study also showed a comparable reduction between 4 and 6 months. Further, the behavioral data showed worsened phenotype at 6 months of age which correlates with the reported muscle necrosis at 6 months of age in SJL/J mice [9], [10]. However, it should be noted that it is difficult to correlate behavioral data with gross histological changes because different techniques were used in different laboratories. Open field testing has not previously been reported. Further, no one has yet performed a time course analysis to correlate behavioral data to the histological change and our study might be the initial step towards achieving that goal.

Fasudil treatment caused an overall decrease in both forelimb and hindlimb grip strength of treated SJL/J compared to untreated SJL/J mice. Fasudil also reduced the horizontal activity of treated SJL/J mice compared to SJL/J controls. In addition, *in vitro* force measurements showed that slow muscle (e.g., soleus) force was decreased by fasudil (although fast muscle (EDL) force was not). Together, the data suggest that the continuous administration of fasudil worsened the disease phenotype in SJL/J mice. The dosage of fasudil (75 mg/kg) used approaches the high end of published data for fasudil efficacy after oral administration. The published doses range from 30–100 mg/kg B.wt [20], [26], [27]. Keeping the broad dose range in mind, 75 mg/kg BW was used for the long-term (16 weeks) study in order to avoid potential toxic effects. Since it is more than double the minimum effective dose used we assumed that it would be sufficient to show an effect. Therefore, it is unlikely that the results reflect inadequate drug levels in the muscle tissue, although we did not specifically measure it. Conversely, the detrimental effect is not likely a result of toxicity, because C57BL6 mice did not show any signs of behavioral deterioration with treatment at the same dosage.

Our rationale for testing fasudil centered on its potential ability to inhibit the downstream activation of ROCK by Rho family GTPases. As Rho-ROCK pathways are activated during phagocytosis and cell motility, then treatment with fasudil, in theory, would reduce the migration of inflammatory cells into muscle. The histological results support this, as monocyte/macrophage infiltration into muscle was reduced by fasudil treatment. Further, this result is consistent with previously observed reductions of F4/80^+^ macrophages, CD4 ^+^, and CD8 ^+^ cells in the glomeruli and the renal cortex of fasudil administered FcR deficient mice [43]. Histological staining of skeletal muscle of fasudil treated vs untreated SJL/J mice indicated no differences in regeneration, degeneration, or muscle fiber injury.

However, it is not clear why fasudil reduced macrophage/monocyte infiltration, but worsened functional outcomes and reduced force strength. Previously, we proposed a model whereby vesicular trafficking, endocytotic proteins, and Rho and Rho family GTPases are up-regulated in LGMD2B patients and SJL/J mice to compensate for dysferlin-deficiency. We also proposed that dysferlin deficiency may lead to decreased expression of immune regulatory molecules, and thus result in overactive immune activation and clearance of damaged myofibers. Reduced macrophage infiltration in fasudil treated muscle clearly indicates that Rok activation plays an important role in SJL/J inflammation. However, because the reduction in macrophages was not associated with improved behavioral outcomes, it raises the possibility that infiltrating macrophages may, at least in part, be beneficial to diseased muscle, and that by limiting macrophage involvement, the disease process is exacerbated. In addition, it is possible that inhibition of ROCK ameliorated the enhanced endocytosis and vesicular trafficking that compensate for dysferlin defiency, and these actions of ROCK might be more important determinants of muscle pathology than inflammatory status. It is also possible that inhibition of ROCK prevented activation of Rok-associated signal transduction cascades that have not previously been considered in the context of dyferlin-deficient myopathy [44], [45]. Notably, ROCK activation induces apoptosis, at least in part, through activation of pro-apoptotic pathways and many studies support that inhibition of ROCK with fasudil prevents cardiomyocyte apoptosis and related dilated cardiomyopathy. Nevertheless, the role of apoptosis, or ROCK, in dysferlin deficient myopathy, has not been well studied, and remains uncertain. Alteration of ROCK activity using the rho kinase inhibitors were reported in multiple different diseases. Further, fasudil has shown beneficial effects in models of disease involving non-dysferlin deficient cardiac muscle [46]–[48]. As rho kinases are involved in multiple pathways it would be difficult to comment if the effects of fasudil observed were specific to dysferlin deficiency

In summary, we present behavioral data that provide a basis for standardization of preclinical drug testing trials in the SJL/J mouse model of dysferlin deficiency. Open field and normalized grip strength data were identified as potentially useful longitudinal, non-invasive measures of functional status, that are potentially translatable to clinical trial outcomes. In addition, the data suggest preclinical drug trials can be initiated and/or performed using the SJL/J model, long before overt muscle damage occurs. Chronic treatment with the Rho-kinase inhibitor, fasudil worsened functional outcomes, but also reduced inflammatory cell infiltrates in diseased SJL/J muscle, which suggests that Rho-kinase may have a multifactorial role in dysferlin-deficient myopathy.

## Supporting Information

Table S1 Comparison of weight, grip strength and rotarod performance of fasudil treated and untreated C57BL6 mice at 9, 17, and 25 weeks of age.
(0.05 MB DOC)

Table S2 Comparison of open field behavior of fasudil treated and untreated C57BL/6 mice at 9, 17, and 25 weeks of age.
(0.05 MB DOC)

Table S3 Histological parameters in the Tibialis Anterior muscle of fasudil treated and Untreated SJL/J mice at 25 weeks of age.
(0.04 MB DOC)
