# Supplementary material for: Characterization of Dysferlin Deficient SJL/J Mice to Assess Preclinical Drug Efficacy: Fasudil Exacerbates Muscle Disease Phenotype
Source: PLoS One. 2010 Sep 24;5(9):e12981. doi: 10.1371/journal.pone.0012981 (PMC2945315; doi:10.1371/journal.pone.0012981)
Supplement: Table S1 — Comparison of weight, grip strength and rotarod performance of fasudil treated and untreated C57BL6 mice at 9, 17, and 25 weeks of age. (0.05 MB DOC) [file pone.0012981.s001.doc]

**Supplementary Table S1:** Comparison of weight, grip strength and rotarod performance of fasudil treated and untreated C57BL6 mice at 9, 17, and 25 weeks of age.

| **Measurement** | **Age (wk)** | **N^1^** | **Treated**  **Mean ± SEM** | **N^1^** | **Untreated**  **Mean ± SEM** | **Power with N=10 per group** |
| --- | --- | --- | --- | --- | --- | --- |
| Weight (g) | 9 | 14 | 21.86 ± 0.36 | 15 | 21.87 ± 0.31^a^ | 5.0% |
|  | 17 | 14 | 30.34 ± 0.43 | 14 | 29.47 ± 0.69^a^ | 14.8% |
|  | 25 | 14 | 35.14 ± 0.60 | 14 | 32.96 ± 0.91^b^ | 39.2% |
| NFL^2^ | 9 | 14 | 4.85 ± 0.09 | 15 | 4.81 ± 0.08^a^ | 5.8% |
|  | 17 | 14 | 3.40 ± 0.13 | 14 | 3.31 ± 0.10^a^ | 7.6% |
|  | 25 | 14 | 3.10 ± 0.09 | 14 | 3.24 ± 0.12^a^ | 12.1% |
| NHL^2^ | 9 | 14 | 11.40 ± 0.16 | 15 | 11.39 ± 0.17^a^ | 5.0% |
|  | 17 | 14 | 8.95 ± 0.24 | 14 | 8.98 ± 0.25^a^ | 5.1% |
|  | 25 | 14 | 8.26 ± 0.15 | 14 | 8.88 ± 0.30^b^ | 35.8% |
| Rotarod (s)^3^ | 9 | 14 | 115.5 ± 6.1 | 15 | 98.8 ± 10.2^a^ | 21.4% |
|  | 17 | 14 | 92.9 ± 5.6 | 14 | 79.0 ± 8.5^a^ | 21.1% |
|  | 25 | 14 | 75.9 ± 4.7 | 14 | 70.4 ± 7.8^a^ | 8.1% |

All data are expressed as mean ± SEM; ^1^ Number of animals per group; ^2^ NFL (Forelimb) and NHL (Hindlimb) grip strength data are normalized to body weight and are expressed in kilogram force per kilogram (KGF/kg); *^*^p*-values are from Wilcoxon rank sum tests at each time point; ^a^Indicates no significant difference between treated and untreated groups; ^b^Indicates significant difference between treated and untreated groups; ^3^Rotarod (s) the length of time in seconds that mice stayed on the rotorod.
