# Supplementary material for: Characterization of Dysferlin Deficient SJL/J Mice to Assess Preclinical Drug Efficacy: Fasudil Exacerbates Muscle Disease Phenotype
Source: PLoS One. 2010 Sep 24;5(9):e12981. doi: 10.1371/journal.pone.0012981 (PMC2945315; doi:10.1371/journal.pone.0012981)
Supplement: Table S2 — Comparison of open field behavior of fasudil treated and untreated C57BL/6 mice at 9, 17, and 25 weeks of age. (0.05 MB DOC) [file pone.0012981.s002.doc]

**Supplementary Table S2:** Comparison of open field behavior of fasudil treated and untreated C57BL/6 mice at 9, 17, and 25 weeks of age.

| Measurement | Age (wk) | N^1^ | Treated  Mean ± SEM | N^1^ | Untreated  Mean ± SEM | *p*-value^*^ | Power with N=10 per group |
| --- | --- | --- | --- | --- | --- | --- | --- |
| Horizontal activity | 9 | 14 | 1086.0 ± 70.2 | 15 | 885.1 ± 55.7 | 0.0447 | 46.3% |
|  | 17 | 14 | 1098.0 ± 64.9 | 14 | 882.6 ± 74.3 | 0.0131 | 45.5% |
|  | 25 | 14 | 1056.0 ± 76.4 | 14 | 1008.0 ± 86.0 | NS^2^ | 6.4% |
| Total distance (cm) | 9 | 14 | 287.1 ± 36.2 | 15 | 194.8 ± 19.5 | NS | 47.0% |
|  | 17 | 14 | 211.6 ± 18.8 | 14 | 152.9 ± 25.5 | 0.0101 | 34.8% |
|  | 25 | 14 | 198.9 ± 32.6 | 14 | 185.0 ± 33.0 | NS | 5.7% |
| Rest time (s) | 9 | 14 | 567.3 ± 3.9 | 15 | 576.4 ± 2.2 | NS | 40.1% |
|  | 17 | 14 | 574.7 ± 2.2 | 14 | 581.4 ± 3.1 | 0.0191 | 30.6% |
|  | 25 | 14 | 576.4 ± 3.7 | 14 | 577.8 ± 3.8 | NS | 5.5% |
| Vertical activity | 9 | 14 | 25.4 ± 3.4 | 15 | 20.5 ± 2.2 | NS | 17.0% |
|  | 17 | 14 | 21.9 ± 2.1 | 14 | 15.7 ± 1.8 | 0.0308 | 48.0% |
|  | 25 | 14 | 19.7 ± 2.7 | 14 | 19.4 ± 2.9 | NS | 5.0% |

All data are expressed as mean ± SEM; ^1^Number of animals per; ^2^NS, Not significant; group; *^*^p*-values are from Wilcoxon rank sum tests at each time point.
