# Supplementary material for: Characterization of Dysferlin Deficient SJL/J Mice to Assess Preclinical Drug Efficacy: Fasudil Exacerbates Muscle Disease Phenotype
Source: PLoS One. 2010 Sep 24;5(9):e12981. doi: 10.1371/journal.pone.0012981 (PMC2945315; doi:10.1371/journal.pone.0012981)
Supplement: Table S3 — Histological parameters in the Tibialis Anterior muscle of fasudil treated and Untreated SJL/J mice at 25 weeks of age. (0.04 MB DOC) [file pone.0012981.s003.doc]

| **Supplementary Table S3:** Histological parameters in the Tibialis Anterior muscle of fasudil treated and Untreated SJL/J mice at 25 weeks of age. | | | |
| --- | --- | --- | --- |
| **Measurement** | **Untreated**  **(*N*=5)** | **Treated**  **(*N*=5)** | ***p*-value^#^** |
| Total number of fibers | 81.69 ± 4.77 | 79.39 ± 2.34 | 0.6734 |
| Degenerating fibers | 0.50 ± 0.28 | 0.41 ± 0.19 | 0.8088 |
| Regenerating fibers | 4.33 ± 0.91 | 3.78 ± 0.60 | 0.6205 |
| Fibers with central nuclei | 29.58 ± 3.47 | 26.72 ± 1.14 | 0.4513 |
| Peripheral nuclei | 192.67 ± 14.69 | 189.69 ± 11.59 | 0.8770 |
| Central nuclei | 36.58 ± 4.40 | 33.19 ± 0.81 | 0.4666 |
| Peripheral nuclei^*^ | 2.43 ± 0.14 | 2.37 ± 0.08 | 0.7457 |
| Central nuclei^*^ | 0.47 ± 0.05 | 0.43 ± 0.01 | 0.4213 |
|  |  |  |  |

All Data are expressed as mean ± SD per field; ^*^Measurement is mean ± SD per fiber; *^#^p* -values are for student’s t-test for independent values.
